# Non-Native Plant Invasion along Elevation and Canopy Closure Gradients in a Middle Rocky Mountain Ecosystem

**DOI:** 10.1371/journal.pone.0147826

**Published:** 2016-01-29

**Authors:** Joshua P. Averett, Bruce McCune, Catherine G. Parks, Bridgett J. Naylor, Tim DelCurto, Ricardo Mata-González

**Affiliations:** 1 Eastern Oregon Agriculture and Natural Resource Program, Oregon State University, La Grande, Oregon, United States of America; 2 Department of Botany and Plant Pathology, Oregon State University, Corvallis, Oregon, United States of America; 3 USDA Forest Service, Pacific Northwest Research Station, La Grande, Oregon, United States of America; 4 Department of Animal and Rangeland Sciences, Oregon State University, Corvallis, Oregon, United States of America; Chinese Academy of Forestry, CHINA

## Abstract

Mountain environments are currently among the ecosystems least invaded by non-native species; however, mountains are increasingly under threat of non-native plant invasion. The slow pace of exotic plant invasions in mountain ecosystems is likely due to a combination of low anthropogenic disturbances, low propagule supply, and extreme/steep environmental gradients. The importance of any one of these factors is debated and likely ecosystem dependent. We evaluated the importance of various correlates of plant invasions in the Wallowa Mountain Range of northeastern Oregon and explored whether non-native species distributions differed from native species along an elevation gradient. Vascular plant communities were sampled in summer 2012 along three mountain roads. Transects (n = 20) were evenly stratified by elevation (~70 m intervals) along each road. Vascular plant species abundances and environmental parameters were measured. We used indicator species analysis to identify habitat affinities for non-native species. Plots were ordinated in species space, joint plots and non-parametric multiplicative regression were used to relate species and community variation to environmental variables. Non-native species richness decreased continuously with increasing elevation. In contrast, native species richness displayed a unimodal distribution with maximum richness occurring at mid–elevations. Species composition was strongly related to elevation and canopy openness. Overlays of trait and environmental factors onto non-metric multidimensional ordinations identified the montane-subalpine community transition and over-story canopy closure exceeding 60% as potential barriers to non-native species establishment. Unlike native species, non-native species showed little evidence for high-elevation or closed-canopy specialization. These data suggest that non-native plants currently found in the Wallowa Mountains are dependent on open canopies and disturbance for establishment in low and mid elevations. Current management objectives including restoration to more open canopies in dry Rocky Mountain forests, may increase immigration pressure of non-native plants from lower elevations into the montane and subalpine zones.

## Introduction

Invasions into natural areas by non-native plant species are considered one of the most significant threats to global biodiversity and ecosystem structure and function [1–3]. Mountain environments are currently among the least invaded ecosystems, however, these biodiversity hotspots are increasingly under threat of non-native plant invasions. Worldwide, over one thousand naturalized non-native species have been identified at high elevations (subalpine or alpine mountain zones), and long established non-native species are moving upwards in some mountains [4–6]. Researchers suggest that the most damaging plant invasions in mountains have occurred recently, and that climate change and shifting anthropogenic land use from agriculture to recreation and tourism may facilitate further spread of non-native plants into high elevations [4,7–8]. Evidence of increasing plant invasion into mountain environments is disconcerting because mountains harbor high biodiversity, contain protected areas, and provide ecosystem services such as storage and delivery of clean water to lower areas [9–10]. Understanding drivers of plant invasion along elevation gradients is important for the development of management and restoration actions that reduce the expansion of non-native plants in mountains.

Because most species encounter distribution limitations along elevation gradients, invasion research in mountains has provided researchers with a unique opportunity to explore non-native species colonization at an invasion front [11]. Two emergent themes in mountain plant invasion research are: 1) non-native species richness decreases strongly at high elevations [6,12–13]; and 2) high elevation, non-native plant communities are typically comprised of generalist species that also occur at the lowest elevations [12,14–15]. Although declining non-native plant species richness at high elevations appears to be a universal trend, low to mid-elevation responses of such species have varied. Most studies, particularly in temperate mountains, have reported continuous declines in non-native species richness with increasing elevation [6,12,14–15]. Research in tropical mountains has revealed hump-shaped responses where non-native richness peaked at mid-elevations similar to mid-elevation peaks for native plant richness [6,16–18].

Native and non-native plant distributions in mountains may be driven by different processes. Native species distributions have been shaped by long histories of evolutionary adaptation to changing biotic and abiotic conditions resulting in high levels of habitat specialization, particularly in high elevation areas where abiotic conditions are harsh and reproductive isolation is common [9,17,19]. Native plant species richness typically peaks at mid-elevations when entire elevation gradients are considered [6,20–21]. The specific mechanisms responsible for native richness patterns have yet to be identified. However, some interaction between 1) environmental gradients [22–23]; 2) habitat heterogeneity [24–25]; evolutionary histories [26]; and 3) geometric constraints, e.g. species area relationships and the mid domain effect [27–28], are thought to account for mid-elevation native richness peaks. Non-native plant distributions are thought to be more strongly influenced by introduction pathways, site preadaptation, and dispersal limitations [12,18,29]. The “directional ecological filtering hypothesis” may explain declining non-native plant richness with increasing elevation [12]. The main idea behind this theory is that historical introduction pathways shape non-native plant distributions in mountain ecosystems. This hypothesis assumes that non-native species are preferentially introduced into low elevation sites. Species pre-adapted to low elevation environmental conditions are able to establish, and generalist species spread into higher elevations while species with narrower environmental tolerances are sequentially filtered out along the elevation gradient [12].

The global similarity of distributional patterns of non-native plant species in mountains implies common causal factors [12]. The dominant factors related to ecological filtering of non-natives along elevation gradients are poorly understood and assumed to change over time [5,12–13,30]. Four major factors that affect non-native plant species abundance and richness with elevation are: 1) climate (temperature and precipitation); 2) disturbance; 3) soil and topographical characteristics; and 4) propagule pressure [13]. These factors are difficult to separate from each other. Several studies have focused on non-native species distributions along mountain roads in attempts to control for disturbance and propagule pressure [16,18,31–32]. Road networks provide excellent conditions for evaluating non-native species distributional responses along elevation gradients due to the efficient seed dispersal they enable and the presence of continuous disturbed habitat along the entire elevation gradient [11,18,33]. Most studies along mountain roads suggest that decreased anthropogenic disturbance and increased climatic stress with increasing elevation interact to filter non-native species along elevation gradients [16,18,31,34–35]. However, less studied factors including propagule pressure, variations in habitat resistance, and changes in soil characteristics with elevation may also influence non-native distributions in mountains [4,18,36–37].

Current patterns of non-native species distributions are directly related to the pool of species available for introduction. Most reported non-native species in mountain environments are early successional, herbaceous species [6,38]. Some research suggests that the paucity of late successional invasive species is less a result of invasion potential and more reflective of past human activities and priorities, e.g. agriculture and pastoralism [39–41]. Increased global trade and shifts in land use from agriculture to recreation and tourism are expected to increase the introduction of high elevation and closed canopy adapted species to mountain environments [4,7,41]. Additionally, range expansion of current invaders into undisturbed areas is expected to increase over time due to climate change and localized species adaptation [5,30]. Several studies have reported rapid local adaptation of introduced species, and time since introduction is often an important predictor for species spread into new and undisturbed areas [5,11,30,42]. Non-native species capable of invading undisturbed habitats, specifically high elevations and closed canopy forests may be particularly destructive to mountain systems as they are not dependent on disturbance for spread [7,41]. Early identification of such species will be critical for successful control in mountain environments.

In the Wallowa Mountains of northeastern Oregon, non-native vascular plant species distributions are thought to be shaped by directional ecological filtering processes [6,12]. However, contrasts in the dominant abiotic factors influencing non-native *versus* native elevation patterns are poorly understood. Our objectives were to evaluate the importance of various correlates of plant invasions in the Wallowa Mountain Range, and to explore whether native species distributions differed from those of non-native species along an elevation gradient. We address the following questions: 1) What habitats are most invaded; 2) How do native and non-native species composition relate to environment and disturbance along the elevation gradient; 3) What are the most important predictors of native and non-native species richness and abundance; and 4) Is there evidence for non-native species specialization to high elevations or closed canopy forests?

## Methods

Field sampling took place on National Forest (Wallowa Whitman National Forest), and private lands. Permission for sampling on public lands was granted by the USDA Forest Service. Private land owner permission was granted for all sites sampled. Field sampling for this study were non-destructive, and did not harm endangered or protected species.

### Study area

Complete understory (vascular plant) community data were collected in summer 2012 in the Wallowa Mountains of northeastern Oregon [43]. The Wallowa Mountains encompass 4,700 km^2^ of steep, deeply dissected topography. The majority of land area in the Wallowa Mountains is publicly owned and administered by the Wallowa Whitman National Forest (USDA Forest Service). Private land ownership is concentrated in the lower elevation valleys and slopes where dominant land uses include cattle ranching, agriculture, and scattered human settlement. Land use at the mid and high elevations includes timber harvest, recreation, and widespread livestock grazing on both public and private lands [44]. Study site elevations range between 902 and 2,264 m. Mean annual precipitation (1981–2010) increases with elevation from 608 to 1,460 mm [43,45–46]. The Wallowa Mountain range is located at the intersection of two climate zones (Temperate Oceanic and Temperate Continental) [47]. The Wallowas experience cool/moist winters and warm/dry summers where approximately 60% of the precipitation (primarily as snow) occurs between November and April and the remaining 40% occurs between May and October. The tendency towards moist winters and dry summers intensifies with decreasing elevation towards the semi-arid valleys [44,47,48]. Mean summer temperatures (1981–2010) range from 11.3 to 19.1°C and mean winter temperatures (1981–2010) range from -5.4 to 0.3°C from the highest elevation sites to the valleys respectively [43,45–46]. Semi-arid bunchgrass and shrub communities are found at the lowest elevations. The mid-elevations support *Pinus ponderosa/Psuedotsuga menziessi* forests on south facing slopes, *Abies grandis/Psuedotsuga menziesii* forests on north slopes, and bunchgrass communities on shallow soil sites. Subalpine forests are dominated by *Abies lasiocarpa*, *Picea engelmannii*, and *Pinus contorta*.

### Field data

Belt transects (n = 60; 20 along each of three roads) were evenly stratified by elevation (~ 70 m intervals) along 97 km of gravel forest roads consistent with the Mountain Invasion Research Network (MIREN) sampling protocol [6]. The three roads included in this survey were Mt. Harris (45°22′ N; 117°53′ W; elevation 902–2046 m), Moss Springs (45°17′ N; 117°45′ W; elevation 1161–2163 m), and Fish Lake (44°56′ N; 117°07′ W; elevation 1051–2264 m) roads [43]. Survey sites along each road traversed lowland, montane, and subalpine vegetation zones [47,49]. Transects were subdivided into three (50 m x 2 m) plots with one plot parallel to the road edge and the other two plots perpendicular to the road plot, together forming a “T” and extending 100 m from the road [6]. Plots were further subdivided into 25 quadrats (2 m x 2 m) for a total of 75 quadrats within each “T” [43]. Vascular plant species abundance were measured within quadrats [43].

All vascular undergrowth (height ≤ 2 m) plants within quadrats were identified to the species level when possible. Several species were lumped by genus (*Carex*, *Juncus*, *etc*.) where specimens lacked phenologic development or structures necessary for identification to the species level. Within each quadrat, percent cover for identified species was classified into one of eight categories: zero (0%); one (> 0 and ≤ 1%); two (> 1–5%); three (> 5–25%); four (> 25–50%); five (> 50–75%); six (> 75–95%); seven (> 95–99%); and eight (> 99–100%) [50]. The use of such cover classes allow for rapid assessment, reduce between observer error, and yield repeatable measurements when a large number of quadrats are sampled [51]. Canopy openness (average of four measurements in the cardinal directions) was measured at 10-m increments along each plot using a spherical densiometer. Disturbance intensity was assessed qualitatively with three categories: one (low); two (moderate); and three (high) to be consistent with MIREN protocols [6]. Low disturbance was defined as no to little visual impact to vegetation or soil from a recent disturbance (affecting < 10% of plot area). Moderate disturbance was recorded when vegetation loss and soil exposure affected 10 to 40% of plot area. High disturbance was recorded where there were signs of removal of vegetation cover and exposure of bare soil on > 40% of plot area.

### Analysis

Analyses were performed using 20 m^2^ subplots (10 m x 2 m). Subplot data were obtained by averaging species abundance (arithmetic midpoint of cover class) for each set of five consecutive quadrats within each plot. Quadrats were combined to reduce noise by improving local estimates of abundance, while maintaining resolution necessary to detect finer-scaled gradients such as distance from roadsides. Ten subplots that were logged less than one week before field sampling were excluded from the data analysis. The remaining species matrix (**A**) consisted of 890 subplots by 385 species prior to transformations and data modifications. Disturbance intensity, habitat type, and slope were assigned to subplots based on the classification of the larger plot in which they were nested [43]. Disturbance intensity (1–3) was used as a quantitative descriptor during the ordination analysis, and as a categorical variable when regression models were used to select important predictors of species richness and abundance.

For ordination analysis, the subplot by species matrix (**A**) was log transformed and species occurring in < 5% of sample units were deleted. We deleted rare species to decrease noise and enhance any signal that related community composition to environmental factors [52]. We log transformed the data to enhance the signal of less frequent species while maintaining monotonicity with the raw data. A generalized log transformation, rather than log(x+1), was used because the smallest non-zero numbers were much less than one: *b* = log(*x*+*x*min)—log(*x*min), where *x* is the raw abundance, *x*min is the smallest nonzero value in the matrix, and *b* is the transformed value. To preserve absolute abundances, no relativizations were used. Multivariate analyses were then performed on a final matrix (**B**) of 890 subplots by 141 species.

#### Biological variables

Subplots were classified into habitat types including: roadside (subplots parallel to roads, located within 2 m of road-adjacent vegetation boundary); grass-shrubland (dominant species included grasses, forbs, and shrubs up to 5 m tall, trees mostly absent); open forest (montane forest with an open canopy and large gaps between trees); closed forest (montane forest where canopy cover was high with few small gaps in the over-story canopy); and subalpine (vegetation over-story dominated by *Abies lasiocarpa*).

We constructed species trait variables to assess their relationships to community gradients. Three traits of interest were: 1) nativity (0 = native, 1 = non-native); 2) abundance potential for a particular species; and 3) observed overall abundance for a particular species. The abundance potential of a species was defined as the maximum percent cover observed in any subplot for that species, and was calculated as the maximum value found within each column of **A**. The observed abundance for each species was defined as the total percent cover observed for that species in all subplots, and was simply the sum total of each column in the species matrix before log transformation. These variables were then combined with other traits into a matrix (**S**). The trait matrix used as an overlay for this analysis was created by multiplying the species matrix (**A**) by the transposed trait matrix (**AS**’).

#### Environmental Variables

To supplement field measurements, climate data were extracted (800 m resolution) from the PRISM model using latitude, longitude, and elevation [45]. Climate variables were then calculated from 30 yr (1981–2010) averages using ClimateWNA for each transect [46]. Derived climate variables include means for spring and summer precipitation, number of frost free days in the spring and summer, frost free period, frost free period start and end dates, temperature difference between the mean warmest and coldest months (continentality), annual precipitation, summer precipitation, and annual maximum and minimum temperatures, as well as extreme minimum temperature over a 30 yr period, and Hargreaves climate moisture deficit. Topographic variables including elevation, aspect index [53], and slope were extracted from digital elevation models (DEM [54]). Elevation was acquired at 60 m resolution at the transect origin along the roadside. We used average slope and distance from road to estimate elevation change at a 2 m resolution consistent with vegetation sampling. Soil variables including available water capacity (fraction of soil water available), available water supply (0–25 cm), percent clay-sand-silt, depth to restrictive layer, and percent organic matter were extracted from the Natural Resources Conservation Service Web Soil Survey [55].

It is important to note that both the climate and soil variables were calculated on a larger scale compared to vegetation measurements. However, we expected to detect broad scale relationships between these variables and community gradients given the large altitudinal gradient and total extent of our study area. We then used finer scale changes in plant communities to infer associations with underlying environmental gradients. Because climate and soil characteristics covary with elevation, it can be hard to separate them from an overall elevation effect. In order to increase our ability to decouple elevation from strong covariates, road selection criteria included selection of roads with similar elevation gradients but as much variation in climate and soil characteristics as possible, e.g. Moss Springs and Mt. Harris roads are located in the northern Wallowas where Temperate Oceanic climate patterns dominate, and basalt is the dominant underlying parent material [47,55]. Fish Lake road is located in the southern Wallowas and experiences more of a Temperate Continental climate, and soil parent materials have a higher composition of granite, argillite, and metasedimentary rocks [47,55].

### Statistical Analysis

#### Invaded habitats

We identified the most common non-native understory vascular plant species based on frequency of occurrence within all subplots and stratified by habitat type. Consistent with our multivariate analysis, we limited this analysis to species that occurred in ≥ 5% of subplots. Because frequency of occurrence does not provide information regarding the dominance of any particular species within a specific habitat, species rank abundances were also plotted for each habitat type. Rank abundances were ordered by total percent cover for each species across all subplots within a given habitat.

Indicator species analysis (ISA) was used to evaluate habitat tendencies for non-native species [56–57]. ISA generates indicator values (IV) that describe the tendencies for species to occur in specific a priori groups, the groups defined in various ways, depending on the goal. IV’s were tested for statistical significance using 10,000 randomizations of group assignments.

#### Relating environment and disturbance to community gradients

Non-metric multidimensional scaling (NMS [57]) using a Euclidean distance measure was used to extract community composition gradients. We used the “slow and thorough” NMS autopilot setting and Kruskal’s strategy 2 for penalization for ties in the distance matrix. NMS was selected because this ordination method is ideally suited to recover nonlinear species responses expected over the large environmental distances sampled [52]. Euclidean distance was used to retain information related to absolute differences in species cover between sample units. Three dimensional ordinations were produced with a random starting configuration and a maximum of 500 iterations. The final configuration was rotated by orthogonal principal axes. Environmental parameters were overlaid on the ordination to investigate relationships between species composition and environmental factors. The ordination was then rotated to load Axis 1 on canopy openness (strongest abiotic correlate with Axis 1) to allow for an easy visual comparison between species traits and environmental parameters. Species traits were overlaid on the ordination to illustrate their relationship to environmental and community gradients.

We evaluated how species distributions were related to the strongest NMS community gradients using nonparametric multiplicative regression (NPMR) in Hyperniche 2.0 [58]. NPMR uses a multiplicative kernel smoother, requiring no assumptions regarding the relationship between response and predictor variables. NPMR automatically models interactions among predictors [59]. Over-fitting protection is provided by leave-one-out cross validation during the model fitting process. We used a local mean estimator, Gaussian kernel, and automatic average minimum neighborhood size option in HyperNiche 2.0 corresponding to 27 for our dataset (the average number of data points bearing on the estimate of the response at each point). Cross-validated *R*^2^ (_X_*R*^2^) was used to evaluate model fit. This differs from the conventional *R*^2^ because it is based on the exclusion of each data point from the estimate of the response at that point. This results in a more conservative estimate of the variability captured by a given model, more closely approximating the true prediction error [60].

We used NPMR response surfaces to estimate optima of target non-native and native species on NMS axes 1 and 2 (community gradients). An initial screening with three-dimensional response surfaces indicated that most species had non-linear relationships and interactions with both NMS axes, i.e. complex single-species patterns. To estimate species optima along each axis, we first needed to control for the interaction with the other axis. To model species optima on Axis 1, we sliced three dimensional responses for each species according to Axis 2 scores. For a given species, this resulted in a two-dimensional plot with Axis 1 scores along the x-axis, species abundance along the y-axis, and multiple response curves corresponding to differing Axis 2 scores. The optimum (highest abundance) response curve along Axis 1 was selected as the distribution of a particular species along that axis as it represented an estimated distribution of that species along Axis 1 when optimal conditions along Axis 2 were met. This procedure was repeated for optima on Axis 2, thus fitting response curves for individual species on each axis while controlling for the other axis [61].

#### Predictors of non-native and native species richness and abundance

We used NPMR to explore relationships of native and non-native species richness to elevation and to identify important environmental and physical habitat predictors of non-native and native species richness and abundance. Models were generated in a stepwise procedure. We evaluated the best models for each response (calculated before deletion of rare species) by balancing the number of predictors and each predictor’s contribution to the overall x*R*^2^ value. A predictor was added to the model if it increased the x*R*^2^ by at least 0.03. Sensitivity was calculated to evaluate the importance of each predictor in a model, based on its influence on species richness and abundance. Sensitivity is the ratio of the relative mean difference in response to the relative mean difference of the predictor, in both cases relative to the range of the variable. For example, if a predictor has a sensitivity of 1.0, we would expect that a 5% change in the predictor would elicit on average a 5% change in the response [59].

#### Non-native high elevation and closed canopy specialization

To investigate the relationship between environmental specialization and invasiveness, we first calculated elevation and canopy openness ranges for each of the 141 species represented in the ordination. Each species elevation range was represented by a lower elevation boundary (2.5^th^ percentile), and an upper elevation boundary (97.5^th^ percentile) calculated from NPMR generated species abundance responses to elevation. We excluded the extreme 5% of each species’ distribution because we considered these as atypical representations of a species range. Canopy openness ranges were calculated in the same fashion.

Species ranges for both elevation and canopy openness were calculated twice to accommodate the fact that species responses to actual elevation may be misleading, because south facing slopes are occupied by lower elevation species and higher elevation communities extend lower on north facing slopes. Likewise, canopy openness measurements fail to capture topographic shading information and fine scale variation and may misrepresent the effective shading at particular subplots. The first range was calculated from estimated species responses to the measured elevation (extracted from DEM), and canopy openness (field measurements). The second range for elevation and canopy openness was calculated from the estimated species response along ordination Axis 2 (strongly correlated to elevation, r = 0.629), and Axis 1 (strongly correlated to canopy openness, r = -0.596) respectively. Because NMS ordination procedures recover the structure of subplots in species space based on species composition information, the order of subplots along ordination axes might better represent the elevation gradient and canopy openness as experienced by species than the actual measured values. Species axis ranges along Axis 2 can be thought of as the “effective elevation” range for a species. Scatterplots of species elevation and canopy openness ranges were examined for evidence of niche specialization.

## Results

### Invaded habitats

A total of 385 understory vascular plant species were recorded within 890 subplots [43]. Approximately 19% (72) of the total understory species pool consisted of non-natives. Of those species, 24 (33%) occurred in at least 5% of the subplots, and were thus retained in the analysis (Table 1).

**Table 1:** Frequency of occurrence by habitat type for non-native plant species. Species indicator values (IV) are reported along with specific habitat tendencies and statistical significance for each species.

Species	Road	Grass Shrub	Open Forest	Closed Forest	Subalpine	Total	% of sites	Habitat Tendency	IV	p-value
*Taraxacum officinale*	158	5	60	11	7	241	27.1	road	34.0	0.000
*Poa compressa*	123	37	32	6	3	201	22.6	grass shrub	48.7	0.000
*Cynoglossum officinale (I)*	94	12	62	29	0	197	22.1	road	13.8	0.011
*Trifolium repens*	98	5	52	10	0	165	18.5	road	14.3	0.003
*Poa pratensis*	81	27	37	8	2	155	17.4	grass shrub	39.4	0.000
*Dactylis glomerata (I)*	96	3	39	3	0	141	15.8	road	22.2	0.000
*Rumex acetosella*	82	0	46	12	1	141	15.8	road	16.4	0.006
*Lactuca serriola (I)*	60	32	36	1	1	130	14.6	grass shrub	53.3	0.000
*Bromus tectorum (I)*	40	44	25	3	0	112	12.6	grass shrub	66.9	0.000
*Thinopyrum intermedium*	65	22	14	6	0	107	12.0	grass shrub	30.7	0.000
*Plantago lanceolata (I)*	85	6	12	2	0	105	11.8	road	14.9	0.000
*Taraxacum laevigatum*	62	1	34	5	0	102	11.5	road	13.4	0.000
*Bromus arvensis*	48	32	19	0	2	101	11.3	grass shrub	56.0	0.000
*Poa bulbosa*	64	18	15	4	0	101	11.3	grass shrub	22.8	0.000
*Tragopogon dubius*	46	22	25	6	0	99	11.1	grass shrub	31.3	0.000
*Ventenata dubia (I)*	45	28	16	0	0	89	10.0	grass shrub	48.4	0.000
*Cirsium arvense (I)*	42	7	26	6	0	81	9.1	grass shrub	3.7	0.415
*Verbascum thapsus (I)*	41	6	25	2	0	74	8.3	grass shrub	6.2	0.033
*Trifolium pratense*	47	4	11	0	0	62	7.0	road	12.8	0.001
*Bromus inermis*	30	0	21	7	1	59	6.6	road	4.6	0.080
*Medicago lupulina*	24	7	22	5	0	58	6.5	grass shrub	5.9	0.034
*Spergularia rubra*	39	4	9	2	0	54	6.1	grass shrub	5.8	0.052
*Bromus brizaeformis*	19	21	5	1	0	46	5.2	grass shrub	38.6	0.002
*Arrhenatherum elatius*	23	1	20	0	0	44	4.9	open forest	4.5	0.046
Number of sites occupied	256	50	170	63	12	551	**61.9**			
Number of sites sampled	300	50	255	185	100	890				
%Habitat occupied	**85.3**	**100.0**	**66.7**	**34.1**	**12.0**					

Notes: “(I) species considered regionally invasive (www.fs.usda.gov/detail/r6/forest-grasslandhealth/invasivespecies)

Non-natives were found most frequently in grass-shrubland habitats (occupied 100% of subplots), followed by roadside (85.3%), open forest (66.7%), closed forest (34.1%), and subalpine habitats (12%; Table 1). Grass-shrubland habitats showed the highest potential to harbor dominant non-native species; six out of the ten most abundant species in such habitats were non-native [43]. *Ventenata dubia* and *Taeniatherum caput-medusae* had the highest rank abundance for all species in grass-shrubland habitats [43]. Non-natives were scarce in closed forest and subalpine habitats where *Thinopyrum intermedium* (rank abundance = 81), and *Taraxacum officinale* (rank abundance = 82) were the most abundant non-native species, respectively [43]. ISA revealed that all but one non-native species had tendencies for roadside and grass-shrubland habitats (Table 1). Only *Arrhenatherum elatius*, a perennial grass widely used for post-disturbance seeding, showed a slight (IV = 4.5, *p* = 0.046) tendency towards open forest sites (Table 1).

### Relating environment and disturbance to community gradients

**Fig 1:**
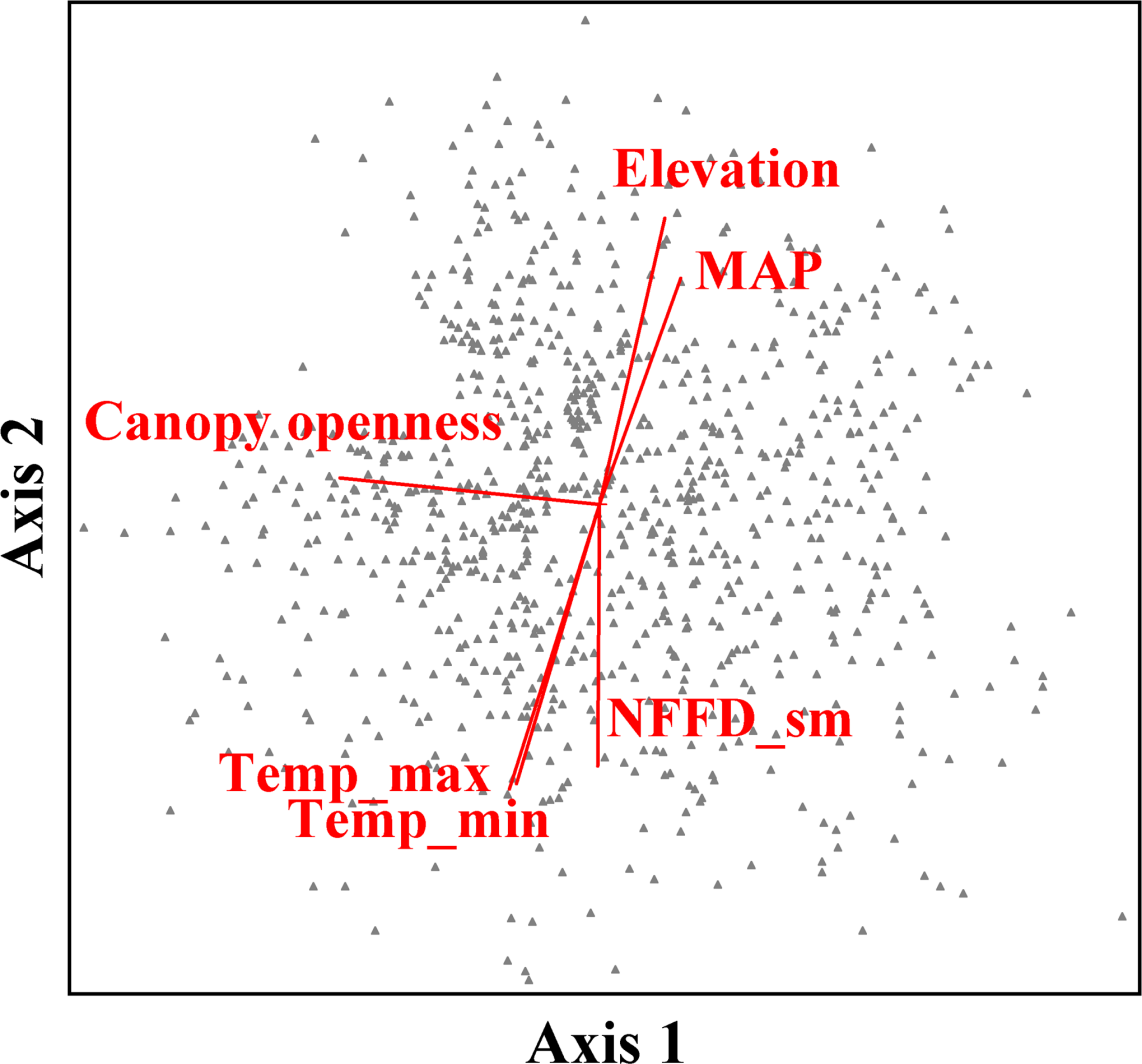
NMS joint plot of Wallowa Mountain plots in undergrowth plant community space. Vectors indicate direction and magnitude (length) of linear correlation between sample units in species space and environmental parameters. MAP (mean annual precipitation), Temp_max (mean annual maximum temperature), Temp_min (mean annual minimum temperature), NFFD_sm (mean number of frost free days in the summer).

The three dimensional NMS ordination (890 subplots by 141 species) yielded a stable solution and represented 74.7% of the variation in the distance matrix (final stress = 17.8; randomization test, *p* = 0.004; Fig 1). Axis 1 explained 31% of community variation and was most strongly related to canopy openness (r = -0.60) followed by soil available water capacity (0.46; Table 2). Along axis 1, a canopy openness gradient was evident where overstory shade increased from left to right along Axis 1 (Fig 1).

**Table 2:** Correlation coeffecients between environmental variables/sample unit traits and NMS ordination axes. Blank cells indicate r ≤ 0.30.

Variable	r	
	Axis 1	Axis 2
Continentality		-0.47
Mean annual precipitation	0.33	0.56
Frost free period start date		0.52
Frost free period end date		-0.45
Frost free period		-0.51
Hargreaves climate moisture deficit	-0.33	-0.53
Canopy openness (%)	-0.60	
Elevation		0.63
Bare-ground (%)	-0.31	
Maximum average temperature	-0.34	-0.62
Minimum average temperature	-0.36	-0.62
Disturbance intensity	-0.42	
AWC (Available water capacity)	0.46	0.44
Clay (%)	-0.45	-0.31
Sand (%)		-0.31
Silt (%)		0.50
Non-native status	-0.64	
Potential abundance	0.40	
Observed abundance	0.67	
Non-native richness	0.61	

Axis 2 (r^2^ = 0.29) represented a strong elevation gradient where elevation (r = 0.63) increased with increasing Axis 2 scores. Temperature and precipitation covaried with elevation along Axis 2. Temperature related variables were negatively related to Axis 2 and precipitation was positively associated with Axis 2 (Fig 1; Table 2).

Axis 3 (r^2^ = 0.15) was most highly correlated with slope (r = 0.40). However, because Axis 3 explained little variation in the distance matrix, and showed primarily weak correlations with species and environmental variables, only Axes 1 and 2 will be discussed further [43].

**Fig 2:**
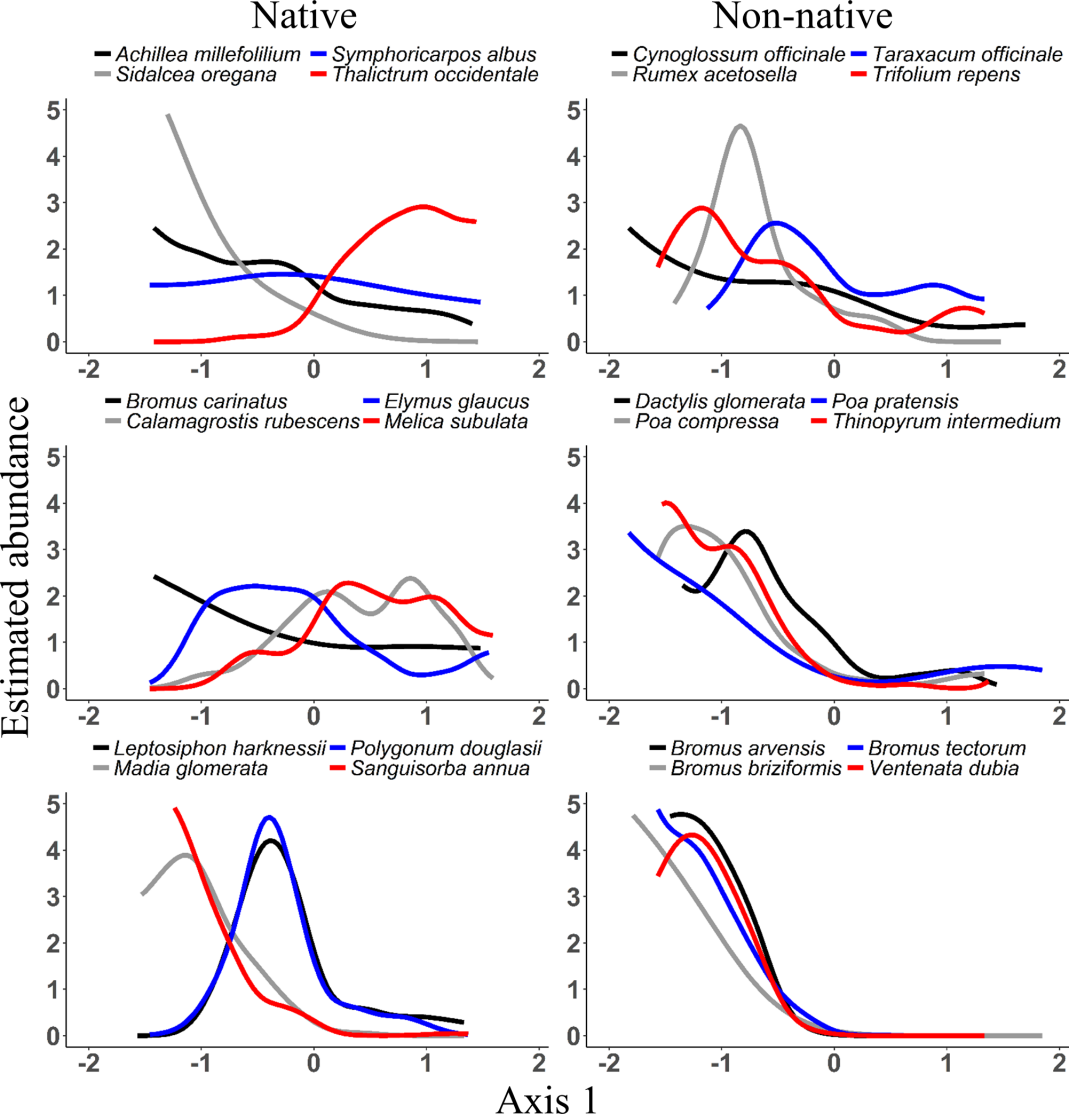
NPMR response curves for selected species along NMS Axis 1 (canopy gradient). Estimated abundances were relativized by the maximum value for each species.

**Fig 3:**
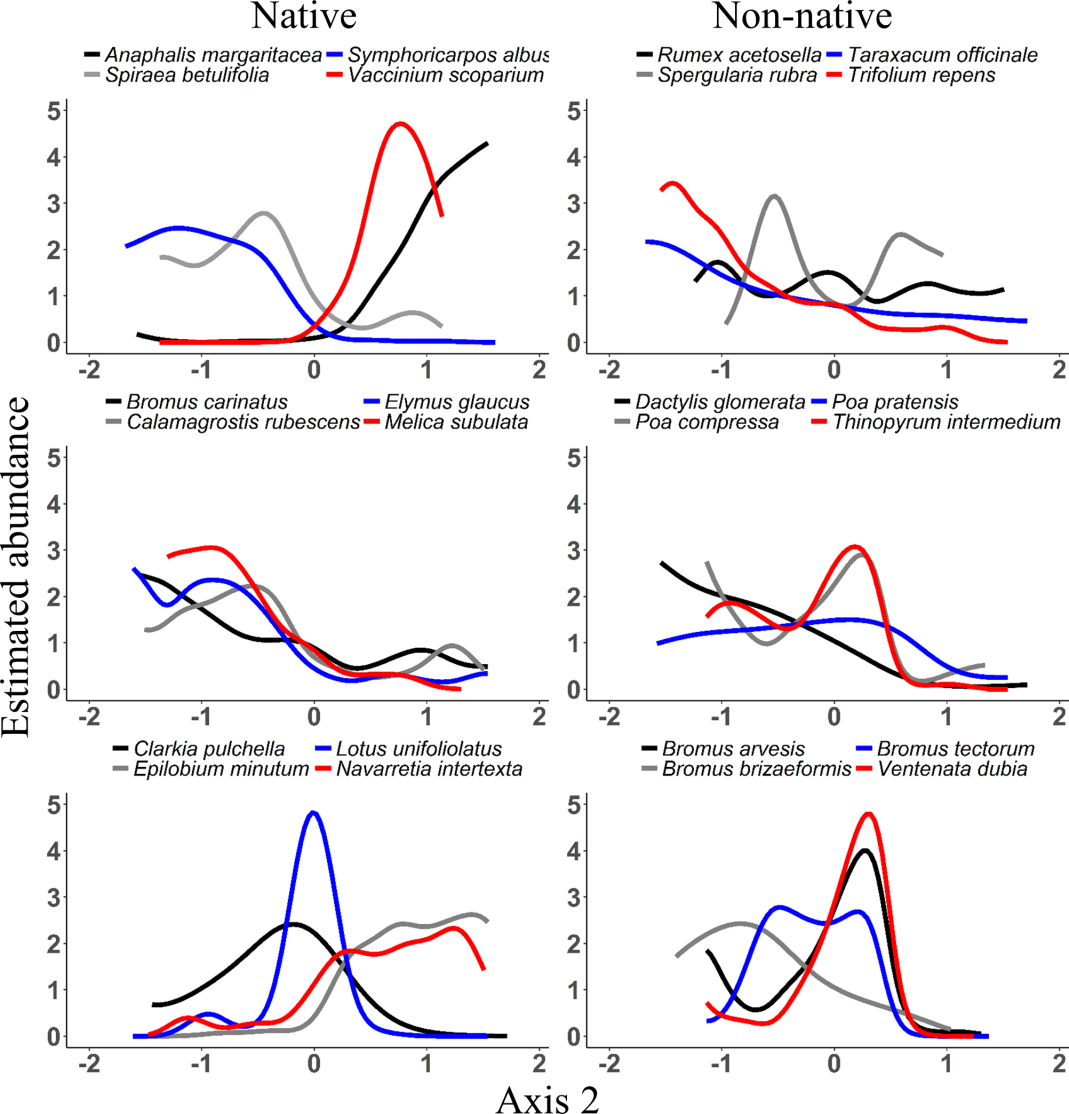
NPMR response curves for selected species along NMS Axis 2 (elevation gradient). Estimated abundances were relativized by the maximum value for each species.

Most species were weakly to moderately associated with the first two ordination axes (NPMR [43]). Cross validated R-squared values ranged from 0.03 (*Cryptantha affinis*) to 0.78 (*Vaccinium membranaceum*). Species abundance responses to ordination axes were variable and nonlinear (Figs 2 and 3).

Non-native species had abundance maxima in sites with open canopies (Fig 2). In contrast, native species showed variable relationships to Axis 1. Early successional, shade intolerant species (*Sanguisorba annua* and *Madia glomerata*) had maxima in relatively open sites, similar to non-natives (Fig 2). Species that favored transitions between open and forested areas (*Leptosiphon harknessii* and *Polygonum douglasii*) were most abundant in sites with low to mid axis scores, and shade tolerant species (*Thalictrum occidentale* and *Melica subulata*) were most abundant in sites towards the dense canopy end of the gradient (Fig 2).

Most (21 out of 24 species) non-natives were negatively correlated with the elevation gradient (NMS Axis 2). After accounting for canopy openness, however, NPMR results showed that non-native species optima along Axis 2 were actually somewhat variable, with maximum abundances in sites with low to moderate scores along Axis 2 (Fig 3). *Rumex acetosella*, and *Spergularia rubra* were the only non-natives not showing substantial declines in abundance with increasing elevation. Responses of native species also varied widely along Axis 2 (Fig 3). Only native species, such as *Epilobium minutum* and *Anaphalis margaritacea*, had maxima high on the elevation gradient (Fig 3).

**Fig 4:**
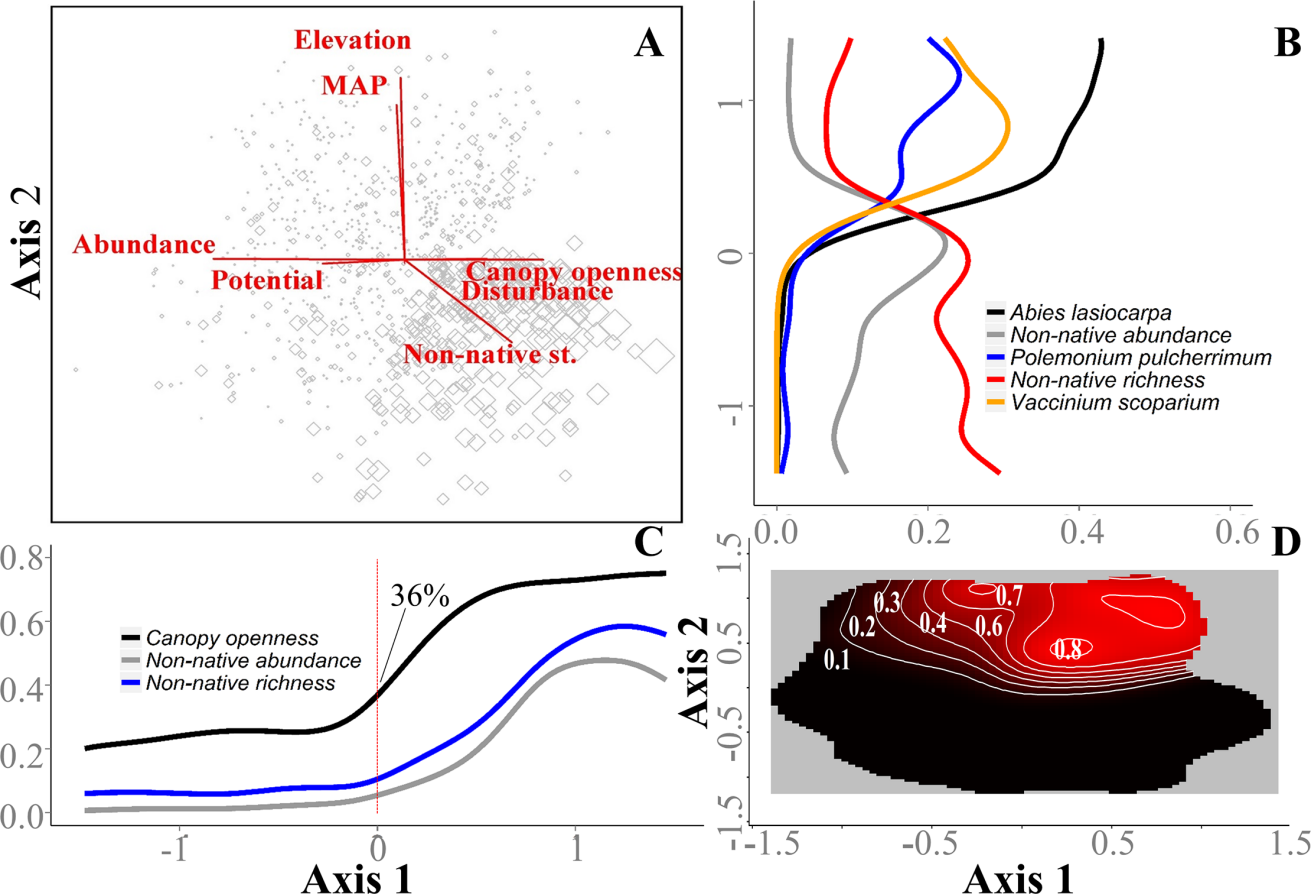
Subalpine community and canopy openness transitions in NMS ordination space. A) NMS ordination of sample units (open diamonds; size corresponds to the abundance of non-native species in each sample unit) in species space (rotated to load canopy openness on Axis 1) and strongly related environmental/trait factors. The combined distance matrix variance represented is 60%, with 30.6% and 29.4% being represented by Axes 1 and 2, respectively. B) subalpine indicator species and non-native richness and abundance responses (NPMR derived) to NMS Axis 2; C) canopy openness and non-native richness and abundance responses (NPMR derived) to NMSAxis 1; B and C) Response variables were relativized by maximum values. D) NPMR derived 3-dimensional contour plot depicting density of subalpine plot sampling in species space. MAP (mean annual precipitation); Non-native st (percent non-native cover relative to total cover); Abundance (observed abundance; sum of cover for a species in all subplots); and Potential (abundance potential; maximum abundance for a species in any subplot).

After rotating the ordination to load canopy openness on Axis 1, non-native species status was positively correlated (0.54) with Axis 1 and negatively correlated (-0.45) with Axis 2, indicating that non-native species were more frequent at low elevation sites with open canopies and moderate to high disturbance (Fig 4A). Species abundance and abundance potential were positively associated with each other and negatively correlated with Axis 1 (-0.700 and -0.452 respectively) indicating that species with high abundance potential were associated with closed canopies and low disturbance (Fig 4A).

Non-native abundance shifts abruptly near the center of the ordination, suggesting potential barriers to non-native species establishment in sites that have negative Axis 1 scores (corresponding to canopy openness < 36%) or positive Axis 2 scores (corresponding to elevations of ≥ 1490 m; Fig 4A–4C). The abrupt decreases of non-native richness and abundance at positive values along Axis 2 appear to correspond to both a paucity of open canopy plots just above the origin along Axis 2 (Fig 4A), and to a transition into subalpine habitat and the lower range of key subalpine indicator species, such as *Abies lasiocarpa* and *Polemonium pulcherrimum* (Fig 4B and 4D). Two dimensional non-linear response curves revealed that non-native species richness and abundance declines coincided with the transition to subalpine habitats along each of the three roads [43].

### Predictors of non-native and native species richness and abundance

**Fig 5:**
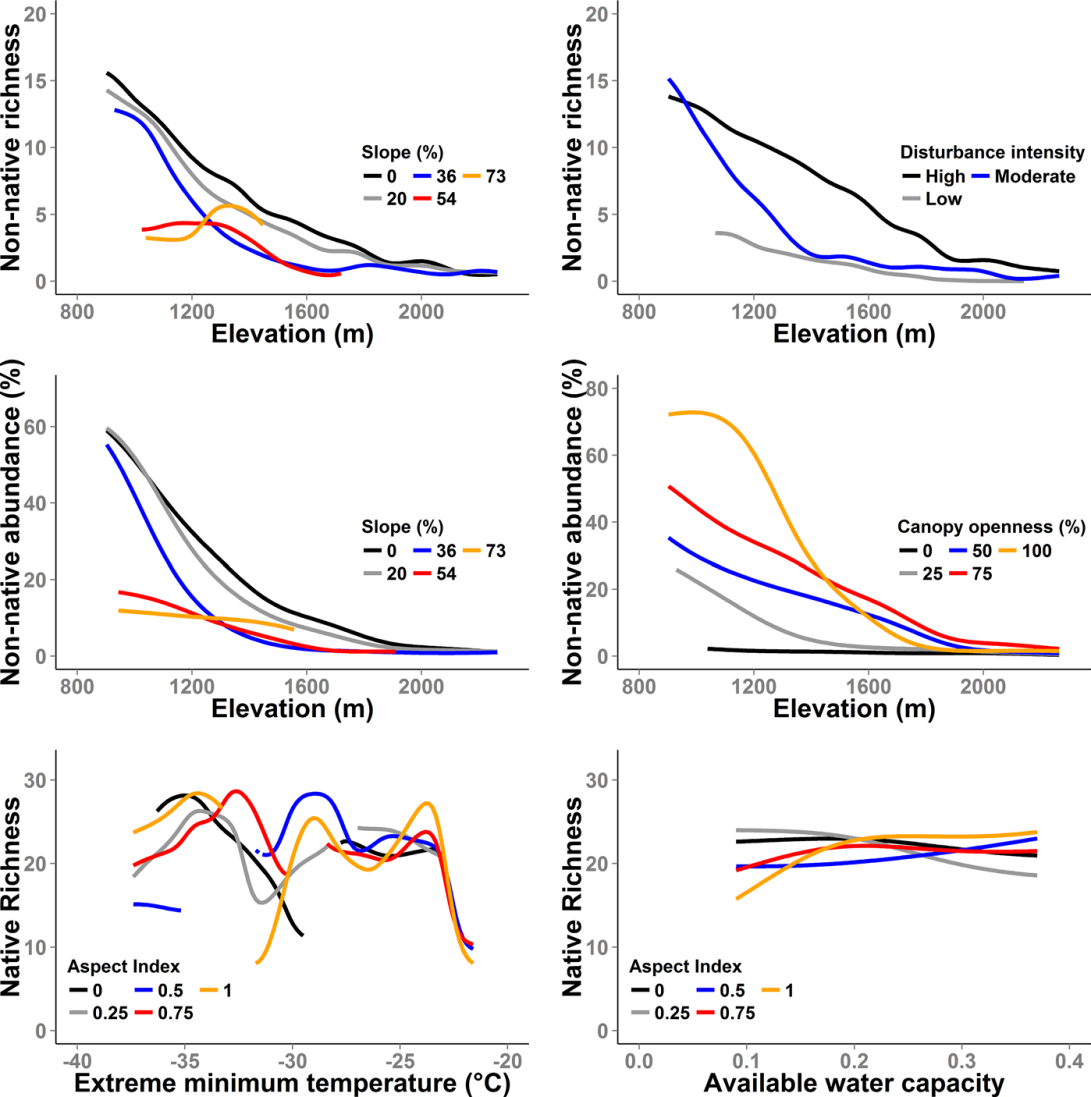
NPMR response curves for the strongest predictors of native and non-native abundance and richness. Non-native abundance is the cover of non-native species relative to total vegetation cover.

Non-native species richness decreased continuously with increasing elevation. Native species richness displayed a unimodal distribution with maximum richness observed at mid-elevations [43]. Elevation (sensitivity = 0.59), percent slope (0.08), and disturbance intensity (0.01) were the strongest predictors of non-native species richness, explaining 75% of its variation, based on the best NPMR model. Elevation was by far the most important predictor. Non-native species richness was inversely related to slope (Fig 5), and increased with disturbance intensity, declining more abruptly with increasing elevation at lower disturbance intensities (Fig 5). Elevation (sensitivity = 0.27) followed by canopy openness (0.22), and slope (0.08) were the best predictors of non-native abundance, and explained 71% of the variation observed. Non-native abundance was inversely related to slope, and increased with canopy openness over most of the elevation range (Fig 5). Extreme minimum temperature over a 30-yr period (sensitivity = 0.43), topographic aspect index (0.139), and available soil water capacity (0.046) were the most important predictors of native species richness. These three factors explained 45% of the variation in native species richness across the 890 subplots sampled. Sliced response curves revealed complex interactions among predictors with respect to native species richness (Fig 5).

### Non-native high elevation and closed canopy specialization

**Fig 6:**
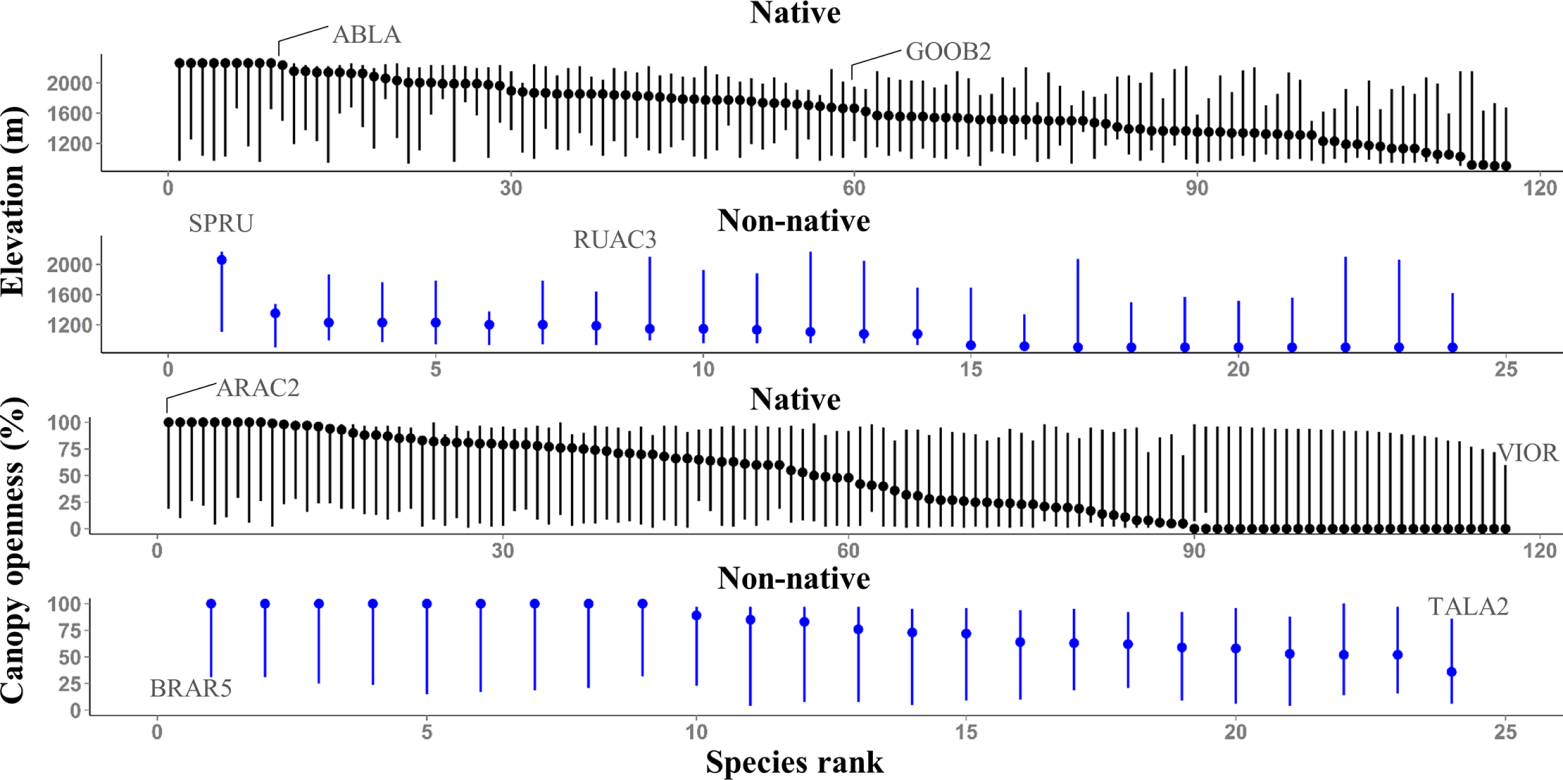
NPMR generated elevation and canopy openness ranges for species (n = 141) included in NMS ordination. Species were ranked by their optima (points) along the elevation and canopy openness gradients. Lines connect between lower (2.5^th^ percentile) and upper (97.5^th^ percentile) range boundaries. ABLA (*Abies lasiocarpa*), ARAC2 (*Arenaria aculeata*), BRAR5 (*Bromus arvensis*), GOOB2 (*Goodyera oblongifolia*), RUAC3 (*Rumex acetosella*), SPRU (*Spergularia rubra*), TALA2 (*Taraxacum laevigatum*), VIOR (*Viola orbiculata*).

**Fig 7:**
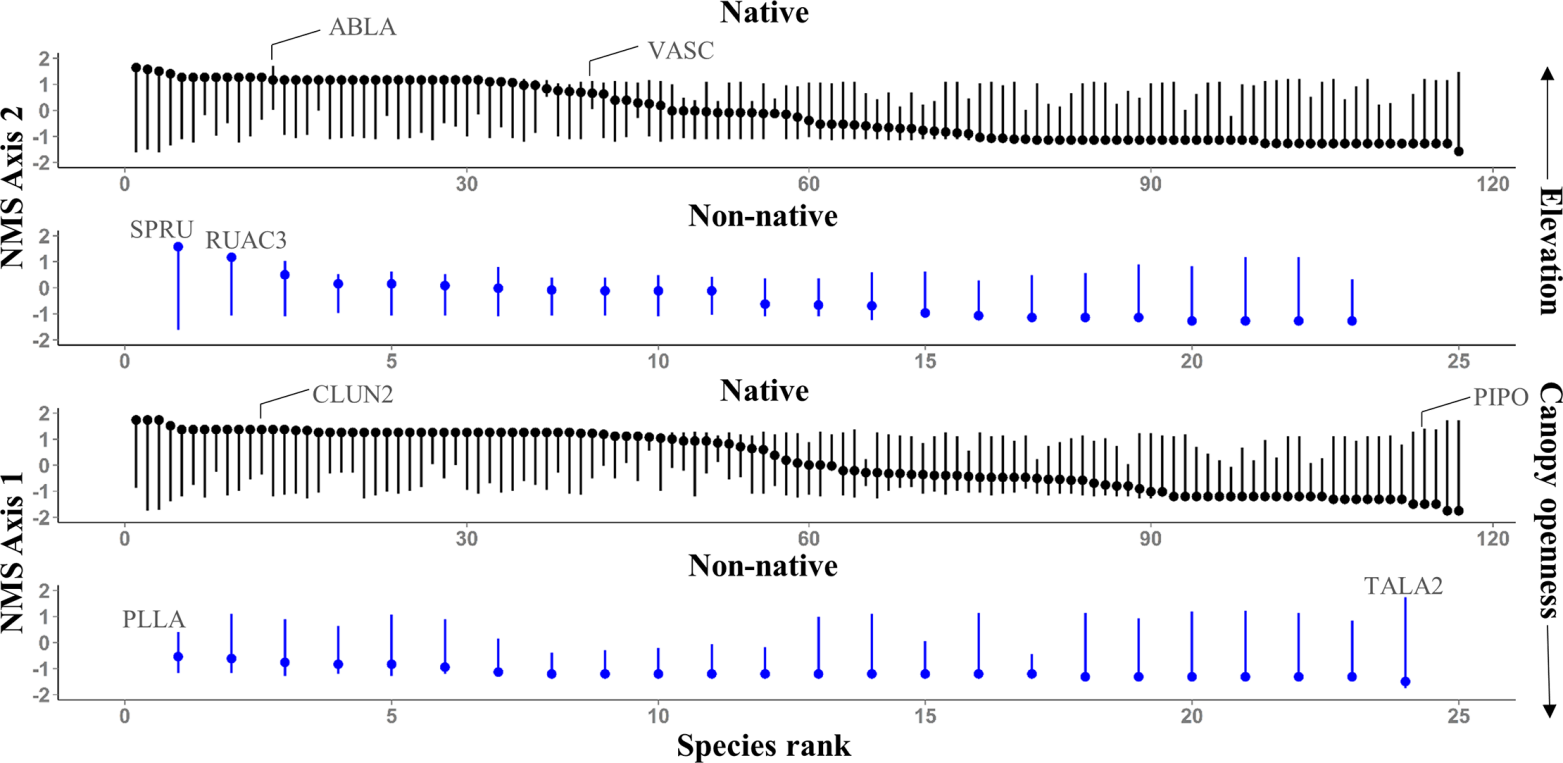
NPMR generated ordination Axes 1 and 2 ranges for species (n = 141) included in NMS ordination. Species were ranked by their optima (points) along each axis. Lines connect between lower (2.5^th^ percentile) and upper (97.5^th^ percentile) range boundaries. ABLA (*Abies lasiocarpa*), VASC (*Vaccinium scoparium*), SPRU (*Spergularia rubra*), RUAC3 (*Rumex acetosella*), CLUN2 (*Clintonia uniflora*) PIPO (*Pinus ponderosa*), PLLA (*Plantago lanceolata*), TALA2 (*Taraxacum laevigatum*).

Non-natives with high elevation occurrences were also present at the lowest elevations (Fig 6). *Spergularia rubra* had the highest (1106 m) lower elevation boundary, and was the only non-native with optimum abundance at high elevations (2060 m; Fig 6). Effective elevation optima for most non-natives ranged from low to moderate values along Axis 2 (elevation gradient; Fig 7). *Spergularia rubra* and *Rumex acetosella* were exceptions, and had optima of 1.6 and 1.2 respectively along Axis 2 both corresponding to greater than 2000 m (Fig 7). High variability of optima and range boundaries for native species revealed that separation of elevational niches were common for natives (Figs 6 and 7). Similar to non-natives, many native species had broad elevation ranges (occurred at both high and low elevations; Figs 6 and 7).

More than half (52%) of native species had optima below 50% canopy openness (Fig 6). In contrast, only one non-native species (*Taraxacum laevigatum*) optimum was below 50% (Fig 6). For both measured and effective canopy openness ranges, native species optima graded from closed canopy (high Axis 1 values) to open canopy sites (low Axis 1 values; Figs 6 and 7). Non-native species optima along Axis 1 ranged from -1.49 to -0.54 corresponding to canopy openness of 67 to 100% (Fig 7). Effective canopy openness ranges for non-native species indicated less shade tolerance when compared to measured canopy openness ranges, which probably reflects finer scale canopy openness not captured with densiometer measurements.

## Discussion

Grass-shrubland habitats had the highest occurrence of non-native species and were the sites most likely to harbor dominant non-natives. The most widespread species did not necessarily show the highest invasion potential. For example, the three most widespread non-natives in our study area (*Taraxacum officinale*, *Poa compressa*, and *Cynoglossum officinale*) were not among the most dominant species in any habitat type. Non-native annual grasses including *Ventenata dubia* (ventenata) and *Taeniatherum caput-medusae* (medusahead) had the highest potential to dominate sites when compared to other non-native species. Annual grass invasions were concentrated in low elevation grass-shrubland sites occurring primarily within fenced cattle pastures. These sites were located along benches (gentle slopes < 20%) above the Grande Ronde River Valley. Historically, these “benchlands” would have likely been dominated by native perennial bunchgrasses including *Pseudoroegneria spicata*, *Festuca idahoensis*, and *Poa secunda* [62]. At present, native perennial bunchgrasses are a minor component of these communities compared to non-native species, accounting for less than 7% of the understory cover in subplots sampled.

Pacific Northwest bunchgrass communities have proven particularly susceptible to annual grass invasions [44,63–65]. Researchers attribute the apparent high invasibility of these systems to early overgrazing coupled with a high influx of pre-adapted non-native winter annual grasses [62–66]. Previous observations revealed that the most heavily disturbed “benchlands” in the Blue Mountain region were initially colonized by non-native winter annual *Bromus* species that were able to out-compete stressed bunchgrasses and their offspring for early season water resources [64–67]. Our findings that ventenata and medusahead were the most abundant species in grassland areas support Johnson and Swanson’s (2005) suspicions that these recently introduced species may have displaced annual *Bromus* species on some sites [64]. Medusahead is thought to be somewhat limited in distribution to sites with heavy clay soil in our region [64]. Our observations support this, as the range of medusahead was constrained to grass-shrubland habitat along Mt. Harris road where high clay content is characteristic of the shallow soil layers [55].

Ventenata was a dominant species in disturbed grass-shrubland and roadside habitats, and was one of the most abundant non-native species in open forest habitats [43]. Anectodal observations suggest that ventenata is currently expanding its range at an alarming rate along road networks and into undisturbed areas [68–69]. Due to its relatively recent introduction (~1956) into the Pacific Northwest, little is known about the potential spread and associated ecological consequences of ventenata in the region [69–70]. Our findings that ventenata has the potential to dominate grass-shrubland and colonize open forests in the Wallowa Mountains suggests that future research should be directed towards understanding the potential invasion dynamics and impacts of ventenata in the Middle Rocky Mountain region.

Previous research suggests that mountain invaders are typically ruderal species that may rely on disturbance for establishment and persistence in mountain environments [7,34,38,71]. We detected a similar pattern, as most non-natives frequented roadsides and disturbed grass and shrublands. However, our results indicated that elevation and canopy openness are the most important correlates with non-native species distributions in the Wallowa Mountains. NMS ordination results indicated potential barriers to non-native species spread along both the elevation and canopy openness gradients. At the low and mid-elevations, canopy openness was the strongest correlate with non-native species abundance. These findings coupled with our inability to identify distance from road as an important predictor of non-native abundance or richness suggests that if over-story canopies are opened at the low to mid elevations, regardless of distance from the road, that non-natives will likely colonize those sites (at least within 100 m from roadsides). We found that disturbance and canopy openness were less important than an overall elevation effect for shaping non-native distributions at the highest elevations. Sparse non-natives despite high disturbance, and exposed bareground in many subalpine sites suggests that either some physical limitation, e.g. propagule dispersal, or abiotic factors, e.g. climate and soil characteristics, rather than disturbance or competitive exclusion by native species are currently limiting non-native establishment in the subalpine zone. An abrupt decline in non-native species abundance and richness was observed midway along the elevation gradient; this corresponded to a paucity of open canopy habitat as well as a transition into the subalpine community. These results suggest several mechanisms that could be responsible for reduced non-native plant invasions in the subalpine zone.

Reductions in immigration pressure at subalpine sites may be at least partially responsible for reduced non-native abundance in subalpine habitats. While propagule pressure was not directly measured, the separation of suitable (disturbed, open canopy) habitat for non-native species at the mid elevations may isolate the subalpine zone. Thus, closed canopies and low disturbance in the montane zone may reduce immigration pressure at higher elevations. While there may be little decrease in propagule pressure along mountain road networks [11,18,33] because roads serve as efficient corridors for seed dispersal of early successional non-native plants, the success of such species depends on the presence of a suitable habitat corridor along the entire length of the road network [33,72]. Our results suggest that the upper montane forest and lower subalpine transition zone may not provide suitable habitat for non-natives due to closed canopy forests and increased topographical shading of roadside habitat.

Although higher elevations probably have lower immigration pressure from non-natives compared to lower elevations in our study area, environmental factors associated with the transition into subalpine communities may be more important for limiting non-natives at high elevations in the Wallowa Mountains. This hypothesis is based on comparable declines of non-native species at the same community transition (subalpine) along all three roads. If dispersal limitations were truly the barrier to high elevation spread of non-native species, we would expect non-native declines to manifest within different vegetation communities along different roads because of variations in geographical distances between vegetation zones, traffic volumes, and disturbance histories.

The occurrence of non-native species only in highly disturbed subalpine plots (roadside and burned areas along Fish Lake Road) indicates that disturbed subalpine sites may be more prone to non-native plant establishment. However, the low richness and abundance of non-natives in burned subalpine sites, despite almost two decades of exposed bare-ground [43], suggest that subalpine sites in the Wallowa Mountains are relatively resistant to invasions by lowland adapted species. These findings are consistent with research that found subalpine and alpine habitats to be substantial barriers to non-native plant spread [13,16,18,73], but contrast with other recent studies that suggest high elevation habitats may be less resistant to non-native plant invasions (given propagule supply and disturbance) compared with lower elevation sites [50, 74]. Cold temperatures, decreased growing season length, soil gradients, and increased precipitation influence transitions from montane to subalpine plant communities in the Wallowa Mountains [47]. We suspect that the same harsh conditions that prevent many native species from surviving in the lower subalpine zone may also limit non-native species.

Predictors of species richness and abundance differed for native and non-native species. Extreme minimum temperature (over a 30 yr period), available soil water capacity, and aspect were most associated with variations in native species richness. In contrast, elevation, canopy openness, and disturbance-related factors were the most important predictors of non-native species richness and abundance. Non-native plants were also favored on gentle slopes. The influence of slope is most likely related to the disturbance history of particular sites. Roadside plots tended to have gentle slopes compared to off-road plots. Additionally, anthropogenic disturbances related to livestock grazing were most concentrated in relatively flat areas including valley bottoms, benches, and ridges where long-term influences on vegetation composition are expected [64].

Niche widths on elevation and canopy openness gradients differed strongly between native and non-native species. Specialization within particular elevation and shade regimes was common for native species, as would be expected for species that have a long evolutionary history in heterogeneous mountain ecosystems. In contrast, non-native species showed little evidence for high elevation or closed canopy specialization. This may suggest that non-native species at high elevations in our study area tended to be generalists also found at low elevations, which may depend on corridors of open canopy habitats for spread up the elevation gradient. Many native species found at high elevations had broad altitudinal ranges similar to non-natives at such elevations. Several native species, including *Carex geyeri* and *Achillea millefolium*, occurred along the entire elevation range and were dominant species in the subalpine zone, indicating that species adapted to low elevations can also have high abundance potential in the subalpine zone. Evidence of elevation optima for the non-natives *Spergularia rubra* and *Rumex acetosella* within high elevation communities suggests that some non-native species introduced at low elevations may actually encounter more suitable conditions as they spread into high elevations. Our failure to find *Spergularia rubra* at the lowest elevations may be a result of incomplete sampling or could illustrate an example of spread from a mid or high elevation introduction. Occurrences of *Spergularia rubra* and *Rumex acetosella* only within roadside plots in the subalpine zone are consistent with other research and suggestive that these species are dependent on highly disturbed sites and open canopies for persistence and unlikely to spread into undisturbed high elevation communities [35,38,75].

Collectively, our results support the directional ecological filtering hypothesis that assumes that non-native species are introduced into low elevation sites by anthropogenic activity, pre-adapted species establish, and species with the widest ecological ranges spread into the highest elevations until they eventually encounter some physiological or other ecological barrier [12]. In our study area, those barriers appear to be the subalpine community and closed canopy forests. Dominant factors responsible for filtering non-native plants along elevation gradients are subject to debate and include: increasingly harsh abiotic factors, e.g. climate and soil characteristics [12,76], decreased propagule pressure [11], decreased disturbance [6], and varying native plant community resistance [74] with increased elevation. Our results suggest that harsh abiotic conditions at the subalpine community transition, and reduced propagule pressure due to isolation of subalpine sites from open canopy habitats at lower elevations by mid-elevation closed canopy forests interact to limit non-native establishment at high elevations in the Wallowa Mountains. Anderson et al. (2015) found that changes in soil characteristics helped to explain non-native plant distributions in the Wallowa Mountains [37]. It is possible that stark contrasts in soil characteristics between the subalpine and montane zones act as a barrier to non-native species. Soil-related barriers may help explain why non-natives in the subalpine zone are more constrained to roadside plots (where road materials originating from lower elevations contrast strongly with high elevation soil materials) compared to the lower elevation zones in the Wallowa Mountains [6,37]. Common trends of decreasing non-native plant richness with increasing elevation and shared non-native flora in temperate mountain ranges around the world suggest that similar mechanisms are acting to shape non-native species distributions in other mountain ecosystems [6,8]. It is likely that non-native plant establishment boundaries are coincident with high elevation community transitions and closed canopy forests in other mountain ranges.

### Management implications

Current land management goals in many Rocky Mountain forests focus on mimicking historic disturbance regimes, including use of prescribed fire and altered logging and grazing practices [77–82]. Desired conditions include shifting forest structure of dry forests within the montane zone from what land managers consider “unhealthy” closed canopy stands of fire intolerant fir (*Abies*) species back into open-park like stands of *Pinus ponderosa* [62,77–78,80–81]. Our findings indicate that canopy closure is an important factor limiting non-native establishment in the montane zone. We expect that the response potentials of many dry montane forests in the Rocky Mountains have been altered with the introduction of non-native species, and that increased canopy openness and disturbance will likely facilitate non-native plant invasions in montane forests as well as provide more connectivity of suitable habitat for non-native species spread into higher elevations. Therefore, the reinstatement of historic disturbance regimes may have the unintended consequence of increasing plant invasions in these systems. We recommend that invasive plant control and monitoring be critical and ongoing components of land management practices that create open canopy conditions in dry montane forests in the middle Rocky Mountain ecoregion. In contrast, subalpine communities may be more resistant to high disturbance events, and will likely require less effort for control of non-native species establishment and spread. We recommend that managers capitalize on the non-native establishment barriers identified in this study (i.e. closed canopies and the montane-subalpine community transition) to buffer subalpine and alpine ecosystems from future plant invasions. Management that focusses on disconnecting suitable habitat (open canopy and disturbed areas) corridors between montane sites and low elevation non-native propagule reservoirs (grass-shrubland and roadside habitat) should decrease propagule pressure into the subalpine zone and reduce the probability of novel adaptation by providing two different evolutionary hurdles for invading plants to overcome (adaptation to both closed canopy and subalpine conditions) instead of one.

## Supporting Information

S1 File Dataset. Species matrix (Table A) where columns include absolute canopy cover of species, and species symbols correspond to USDA Plant Database (plants.usda.gov) practices. Environmental matrix (Table B). Column A includes subplots (Tables A and B): FL corresponds to Fish Lake road; MH corresponds to Mt. Harris road; and MS corresponds to Moss Springs road. Species list (Table C) with corresponding symbols.
(XLSX)
